# Defective Mitochondrial Fatty Acid Oxidation and Lipotoxicity in Kidney Diseases

**DOI:** 10.3389/fmed.2020.00065

**Published:** 2020-03-12

**Authors:** Hee-Seong Jang, Mi Ra Noh, Jinu Kim, Babu J. Padanilam

**Affiliations:** ^1^Department of Cellular and Integrative Physiology, University of Nebraska Medical Center, Omaha, NE, United States; ^2^Department of Anatomy, Jeju National University School of Medicine, Jeju, South Korea; ^3^Interdisciplinary Graduate Program in Advanced Convergence Technology & Science, Jeju National University, Jeju, South Korea; ^4^Internal Medicine, Section of Nephrology, University of Nebraska Medical Center, Omaha, NE, United States

**Keywords:** mitochondria, fatty acid β-oxidation, lipotoxicity, acute kidney injury, chronic kidney disease, diabetic nephropathy, polycystic kidney disease, glomerular nephropathy

## Abstract

The kidney is a highly metabolic organ and uses high levels of ATP to maintain electrolyte and acid-base homeostasis and reabsorb nutrients. Energy depletion is a critical factor in development and progression of various kidney diseases including acute kidney injury (AKI), chronic kidney disease (CKD), and diabetic and glomerular nephropathy. Mitochondrial fatty acid β-oxidation (FAO) serves as the preferred source of ATP in the kidney and its dysfunction results in ATP depletion and lipotoxicity to elicit tubular injury and inflammation and subsequent fibrosis progression. This review explores the current state of knowledge on the role of mitochondrial FAO dysfunction in the pathophysiology of kidney diseases including AKI and CKD and prospective views on developing therapeutic interventions based on mitochondrial energy metabolism.

## Introduction

The kidney demands a high energy supply to generate energy-required transport of glucose, ions, and nutrients from blood filtrate (1). Mitochondria is an essential organelle generating ATP through oxidative metabolism, as well as regulation of redox homeostasis and cell death signaling. Loss or depletion of ATP by renal tubular mitochondrial disturbance causes acute and chronic kidney diseases (2–5). Renal tubules, particularly proximal tubules that have abundant mitochondria, are metabolically active due to reabsorption of most glomerular filtrate. Because medullary region with pars recta adjunct proximal tubule has only 5–10% of total renal blood flow with tissue oxygen tension 10–20 mm Hg, the medullary proximal tubules are highly vulnerable to hypoxic condition such as ischemia/reperfusion injury (6, 7). Mitochondrial fatty acid β-oxidation (FAO) in proximal tubule is a major source of ATP generation, and its impairment is linked to ATP depletion-induced acute kidney injury (AKI) (1), lipotoxicity (8, 9), and its long-term sequelae leading to CKD (10). Several reports demonstrate that AKI is an independent risk factor for CKD (11–15), and thus promoting mitochondrial FAO is a first-rate option for preventing AKI and CKD. Recent reports indicate that podocytes are also highly sensitive to acute and chronic stimuli, because podocytes have a limited ability for adaptation to mitochondrial energy crisis (16). Here, we summarize the recent findings associated with mitochondrial dysregulation, particularly defective fatty acid (FA) metabolism and lipotoxicity in kidney diseases, which includes tubular and glomerular injury. We also discuss therapeutic strategies targeting mitochondrial energy metabolism in kidney diseases.

## Mitochondrial Energetics

The mitochondria are highly dynamic intracellular organelles that generate most of the ATP in tissues, including the kidney (17). The kidney has abundant mitochondria to produce high levels of ATP through oxidative phosphorylation, to accomplish the substantial passive or active reabsorption of components of the glomerular filtrate, including various ions, glucose, and nutrients. Oxidative phosphorylation yields 36 ATP per glucose, which is highly efficient compared to that of glycolysis generating only 2 ATP (18). The most efficient ATP-generating system in cell energy metabolism is FAO, which can generate 106–129 ATP, depending on the number of carbons in the FA chain. The proximal tubule transports ~67% of glomerular filtrate and thus requires high levels of ATP for its function (19). The proximal tubule prefers FAO for ATP production and has low metabolic flexibility to glycolysis (6, 20). Moreover, it should be noted that the outer medullary proximal tubule has lower oxygen tension under normal conditions and thus less capacity to cope with hypoxic condition (6), which makes them highly sensitive to acute and chronic stimuli. On the other hand, distal tubule is less susceptible to acute stimuli such as ischemic injury and nephrotoxins, because it has better capacity for glycolytic adaptation during hypoxic/ischemic condition, despite its high energy requirement (6, 21, 22). The glomerular podocyte, which has less mitochondria than proximal tubule and depends on mitochondrial respiration for ~75% of energy, also has high vulnerability to stimuli such as glycemic condition (16), but the mechanism of its susceptibility remains to be fully defined. In diseased kidneys with impaired FAO, glycolysis and glutaminolysis can serve as a significant energy source. For example, in polycystic kidney disease (PKD), metabolic reprogramming by increased glutaminolysis, as well as glycolysis, occurs to cope with impaired FAO (23). However, in the ischemic kidney, it has been reported that poly (ADP-ribose) Polymerase 1 and Tp53 induced glycolysis and apoptosis regulator are selectively activated in the injured proximal tubules and inhibit glycolysis during ischemic injury (24, 25). This will prevent compensation of ATP production by glycolysis and makes the proximal tubules extremely vulnerable. Intriguingly, recent reports suggest upregulation of glycolysis as a compensatory mechanism to adapt to reduced FAO during persistent acute tubular injury, which may be related with tubular repair mechanism, resulting in chronic inflammation and fibrosis progression (26–28). These studies indicate that adaptation of energy metabolism for loss of mitochondrial ATP could be compensated by other metabolic processes such as glycolysis or glutaminolysis, suggesting that regulatory mechanism of metabolic pathways can be a key to develop a valuable target for treatment of kidney diseases.

## Mitochondrial Fatty Acid Metabolism

**Figure 1:**
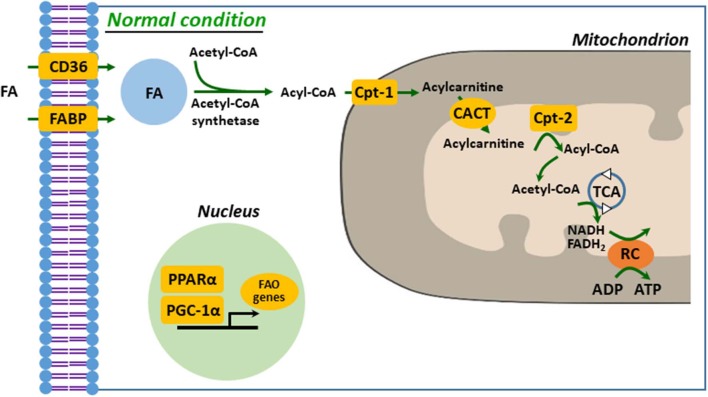
Mitochondrial fatty acid oxidation in kidney tubule. FA enters into cytosol of renal proximal tubule cell (PTC) via FABP or CD36. In the cytosol, FA are converted from acetyl-CoA to acyl-CoA by acetyl-CoA synthetase and then transferred to mitochondrial matrix by carnitine shuttle, Cpt-1, CACT, and Cpt-2, step by step. Acyl-CoA undergoes β-oxidation to produce acetyl-CoA for TCA. NADH and FADH2 generated by TCA are used as electron donors for RC. FA, fatty acid; FAO, fatty acid β-oxidation; Cpt, carnitine *O*-palmitoyltransferase; CACT, carnitine-acylcarnitine translocase; TCA, tricarboxylic acid cycle; RC, respiratory chain.

Defective FA uptake, synthesis, and oxidation are tightly linked to development and progression of kidney diseases. In proximal tubular cells, FA can be taken up by membrane FA transport proteins, such as CD36 and FA-binding protein (FABP), as well as by reabsorption from glomerular filtrate by endocytosis of receptor-mediated FA-bound albumin (20, 29, 30). The kidney with AKI accumulates FAs in cytoplasm, which is a result of dysregulated FAO, leading to ATP depletion (1). Fatty acid is activated to acyl-CoA to make it permeable to the outer mitochondrial membrane (OMM) by acyl-CoA synthetases in the cytosol. Carnitine palmitoyltransferase-1 (Cpt-1), located on the OMM, catalyzes transesterification of the acyl-CoA to acylcarnitine (20, 21, 31). Acylcarnitine is shuttled across the inner mitochondrial membrane (IMM) through carnitine–acylcarnitine translocase. Acylcarnitine is reconverted to acyl-CoA by Cpt-2, an IMM protein. In the mitochondrial matrix, through β-oxidation, a serial cyclic process is trimmed the acyl-CoA to form molecules of acetyl-CoA (21). Finally, acetyl-CoA is fed into the tricarboxylic acid cycle, to generate NADH and FADH_2_ that serve as electron donors to the electron transport chain for ATP production (20, 29, 31) (Figure 1). It is well-known that downregulated or deficient CPT-1 or CPT-2 is critical to impaired FAO in diverse kidney diseases, such as ischemic and cisplatin AKI and diabetic nephropathy (32–34).

**Figure 2:**
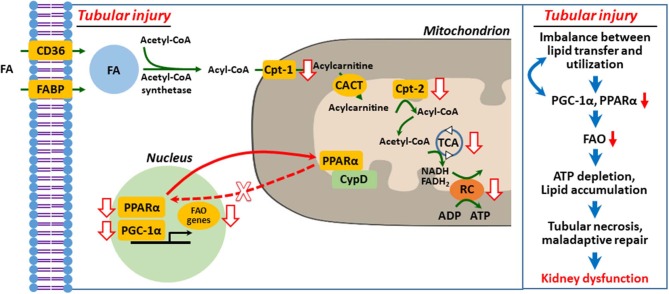
Defective mitochondrial fatty acid oxidation and lipid accumulation in injured kidney tubular cell. Upon tubular injury, PPARα translocates to mitochondria and binds with cyclophilin D (CypD), resulting in mitochondrial sequestration and decreased transcriptional activity of PPARα for FAO genes (38). Inhibition of FAO genes depletes ATP by impaired FAO and that in turn induces PTC necrosis, maladaptive repair, and kidney dysfunction (1, 9, 20, 29, 36, 38, 39, 43, 44). FA, fatty acid; FAO, fatty acid β-oxidation; Cpt, carnitine *O*-palmitoyltransferase; CACT, carnitine-acylcarnitine translocase; TCA, tricarboxylic acid cycle; RC, respiratory chain.

It is well-recognized that peroxisome proliferator-activated receptor γ coactivator-1α (PGC-1α)–peroxisome proliferator-activated receptor α (PPARα) axis governs transcription and regulation of FAO genes in diverse tissues, including the kidney, and its regulation has been suggested as a therapeutic target of AKI and CKD (10, 20, 35–37). We (38) and others (39–42) suggested that defective mitochondrial FAO is critical to ischemic and cisplatin-induced AKI (Figure 2). Downregulation of activity and expression of PPARα and/or PGC-1α resulted in inhibited transcriptional regulation of FAO genes, such as Cpt-1 and medium chain–specific acyl-CoA dehydrogenase, leading to decreased mitochondrial FAO (38, 40, 45). Enhanced PPARα activation by fenofibrate protects histological and functional impairment in cisplatin AKI (46). However, the upstream signaling pathway that inhibits PPARα-regulated FAO in AKI is under investigation.

In our recent report (38), we hypothesized that mitochondrial interaction of proximal tubule cyclophilin D (CypD) and PPARα modulates FAO in cisplatin AKI. We demonstrated using genetic and pharmacological intervention, protein–protein interaction studies, and bioinformatics that mitochondrial CypD-PPARα binding, which modulates FAO, occurs in proximal tubule during cisplatin AKI. Mitochondrial translocation of PPARα, its binding to CypD, and sequestration led to inhibition of its nuclear translocation and transcription of PPARα-regulated FAO genes during cisplatin AKI, leading to reduced FAO, lipid accumulation, and lipotoxicity. Pharmacological or genetic inhibition of CypD promoted nuclear translocation of PPARα and enhanced the transcription of FAO genes and prevented cisplatin AKI (Figure 2).

In CKD, the PGC1α-PPARα axis and FAO key enzymes such as Cpt-1 are persistently decreased. In hypertensive and folic acid–induced CKD, tubular lipid accumulation related to defective FAO, along with tubular and functional impairment, is reported (10, 47). In proximal tubule exposed to FA, PPARα activation can eliminate ceramides, which are toxic metabolites contributing to lipotoxicity (20, 43, 48). On the other hand, in addition to its role in FAO, PGC-1α can act as a master regulator of mitochondrial biogenesis and NAD biosynthesis. This topic is reviewed in detail elsewhere (1, 8, 36, 37, 49).

## Glomerular/Podocyte Injury

Glomerular podocytes are specialized epithelial cells, integrating structural and functional maintenance of glomerular filtration barrier. Podocytes consume high energy for their function, which makes the cells highly susceptible to ATP depletion and to acute and chronic injury (16, 50, 51). Podocyte injury and loss contribute to initiation and progression of proteinuric glomerular diseases, including diabetic nephropathy, glomerular sclerosis, and membranous nephropathy (51–53). In hyperglycemic condition, mitochondrial FAO is enhanced in podocytes, but when both FAO and glycolysis were inhibited, it has a limited capacity to adapt to the changed condition and sensitize podocytes to apoptosis (16, 54). In addition, because mitochondrial respiration accounts for 75% of energy, podocytes have less glycolytic flexibility when mitochondrial function is impaired, resulting in energy deficit (16, 55). However, because glomerulus consists of other cells, including mesangial cell and endothelial cells, all of which could interact with podocytes, in diseased conditions, data from *in vitro* studies using only podocytes should be interpret cautiously (16). On the other hand, it seems that PPARα activation impedes the progression of diabetic nephropathy. PPARα is increased in the kidneys of streptozotocin-induced diabetic mouse model, but when PPARα was genetically deleted, the mice showed adverse effects, including glomerulosclerosis (56). Similar findings from types 1 and 2 diabetic animal models demonstrated that activation of PPARα by fibrates improved hyperglycemic and/or dislipidemic condition–induced glomerular injury and function along with lipid lowering effect (56–60). Although human data (61) revealed that fibrates improve diabetic nephropathy such as albuminuria, data from rodent studies demonstrate more effectiveness than those of clinical studies, suggesting that rodents are more sensitive to PPARα signaling. These data suggest that caution is needed to interpreting the effectiveness of fibrate treatment from animals to humans (61, 62).

## Genetic Disorders

Many genetic disorders are involved in initiation and progression of kidney diseases. Polycystic kidney disease due to mutations in PKD1 and PKD2, which produces polycystin 1 and 2, respectively, are the most common monogenic human kidney diseases, showing 100–1,000 fluid-filled renal cysts (63). A number of signaling pathways, including cAMP, calcium, cell cycle, mTORC1, and WNT signaling, are involved in PKD pathogenesis (63, 64). Recent reports demonstrated defective FAO, as well as glucose metabolism, can contribute to the pathogenesis of both human and animal autosomal dominant PKD (ADPKD) (64, 65). Polycystin proteins seem to be involved in mitochondrial function, because epithelial Pkd1 inactivation from proximal or distal tubule resulted in lower FAO with unchanged glycolysis (66, 67). It is reported that loss of Pkd1 drives cyst growth with declined FAO via direct repression of PPARα (23, 66). Upregulation of PPARα by fenofibrate enhanced FAO and showed beneficial effect in slowing PKD progression by suppressed renal cyst growth, fibrosis, and improved function in a slowly progressing orthologous model of ADPKD (68). On the other hand, the role of FAO in autosomal recessive PKD, a recessive form of PKD that is a rare genetic disorder characterized by enlarged kidney and biliary dysgenesis (63, 69), remains largely unknown.

## Lipotoxicity

Although the causal relationship is unclear, a number of reports suggest that lipid accumulation in certain tissue and cell could be harmful and is referred to as lipotoxicity (43, 70, 71). The initial hypothesis regarding lipotoxicity was that intrarenal lipid accumulation can affect structure and function in renal cells, including proximal tubule cell (71, 72). Accumulation of triglyceride, which is produced by dysregulated glycerol and non-esterified FA (NEFA) presumably derived from impaired FA transport and/or FAO in cytoplasm causes lipotoxicity, contributing to decreased production of ATP and mitochondrial energy metabolism (43, 44). NEFA triggers mitochondrial dysfunction as a cause of energetic failure of proximal tubules during hypoxia/reoxygenation, and intracellular accumulation of NEFA and triglycerides with downregulation of mitochondrial FAO (43, 73). Accumulation of triglycerides is observed in tubule injured by ischemic, cisplatin, glycerol-induced, and septic AKI, as well as in kidneys with metabolic syndrome or fibrosis progression (10, 44, 71, 74). Lipid accumulation in ischemic proximal tubule may result in persistent energy depletion with NEFA-induced mitochondrial dysfunction (43). In parallel, high-fat diet or palmitic acid overload resulted in upregulation of inflammation, fibrosis, or cell death in kidneys (75–77). However, it is still under debate whether FA or triglyceride *per se* is toxic, but it is clear that intrarenal lipid accumulation, by as of yet undefined mechanisms, can represent characteristics of diseased status (43, 70, 78). Recent data show that in two CKD mouse models (diabetic nephropathy and folic acid nephropathy) lipid accumulation by kidney cell–specific overexpression of CD36, a key membrane protein for FA uptake in proximal tubule (79, 80), did not generate renal fibrosis (10). It is proposed that mitochondrial defects in energy production are more detrimental than the lipid accumulation in the cytoplasm. Further studies to define the causal relationship between lipid accumulation and energy depletion and the effect of lipotoxicity during AKI and CKD are warranted.

## Targeting Mitochondrial Fatty Acid Metabolism in Kidney Diseases

A number of studies targeting mitochondrial dysfunction in kidney diseases have been investigated in both animals and human (29). The most treatable option targeting defective FAO in AKI and CKD to date is using agonists of PPARα, fibrates–fenofibrate, clofibrate, and others, despite its adverse effects (81, 82). Fibrates showed a preventive effect to tubular cell death and dysregulated intracellular lipid accumulation, in ischemic and cisplatin AKI models, and in high-fat diet or folic acid–induced CKD models (46, 83–86). However, fenofibrate treatment has adverse effects in kidney function as demonstrated by decreased glomerular filtrate rate and/or increased serum creatinine independent of its lipid-lowering effect (82, 87–89). These data suggest that a better understanding of the molecular mechanism of PPARα agonists and its tissue specificity is required to assess the effectiveness of fenofibrate therapy. Another promising option to modulate FAO is to target FA synthase or transporter. Administration of 5A peptide, which targets CD36 to inhibit FA transport into cell, showed promising results by lowering intrarenal lipid level in subtotal nephrectomized mice kidneys (90). Like CD36 antagonist, a blocker of FA synthase, C75, showed beneficial effect in suppression of folic acid–induced kidney fibrosis progression (10).

Other treatment strategies targeting mitochondria, but not targeting FAO *per se*, include the use of SS-31 (Szeto-Schiller 31) and MitoQ or MitoT. SS-31, mitochondria-targeting tetrapeptides, preserved mitochondrial structure in both proximal tubules and podocytes and thus enhanced functional recovery from ischemic AKI and prevents its long-term consequences, including interstitial fibrosis and glomerulosclerosis (91, 92). In high-fat diet–mediated proximal tubule injury, SS-31 lowered intracellular lipid accumulation by suppressing disruption of mitochondrial function (93). Mitochondria-targeted lipophilic antioxidants, MitoQ and MitoT, protected tubular injury and kidney dysfunction through suppression of mitochondrial damage and oxidative stress and improvement of mitochondrial NADPH level in septic or cisplatin AKI (94, 95). One of the major barriers to develop treatment strategies targeting mitochondrial dysfunction in AKI and CKD is to take into consideration that mitochondria is an organelle regulating redox homeostasis by reactive oxygen species production and detoxification, and its dysregulation could increase oxidative stress (8, 96). Thus, an integrated understanding for mitochondrial biology, including mitochondrial energy metabolism and redox signaling, in particular in susceptible kidney segments, should be preceded to minimize the side effects of mitochondrial targeting in kidney diseases.

## Conclusion

Mitochondrial dysregulation, resulting in loss of ATP, is critical to energy homeostasis and pathogenesis of kidney diseases. Acute and chronic disturbance of mitochondrial FA metabolism depletes ATP, leading to tubular and glomerular injury. Lipotoxicity via impaired FA metabolism could induce cell death and inflammation and promote the chronic progression of AKI to CKD. Unveiling the role and the related molecular mechanism of mitochondrial energy metabolism is required for the development of effective therapeutics in targeting tubular and glomerular injury in acute and chronic kidney diseases.

## Author Contributions

H-SJ and BP made substantial contributions to the conception and drafting the work and revising it critically for important intellectual content, provided approval for publication of the content, and agreed to be accountable for all aspects of the work in ensuring that questions related to the accuracy or integrity of any part of the work are appropriately investigated and resolved. MN and JK made contributions to drafting the work or revising it critically for important intellectual content and provided approval for publication of the content.

### Conflict of Interest

The authors declare that the research was conducted in the absence of any commercial or financial relationships that could be construed as a potential conflict of interest.
